# Using the Expanded Andersen Model to Determine Factors Associated with Mexican Adolescents’ Utilization of Dental Services

**DOI:** 10.3390/healthcare11243159

**Published:** 2023-12-13

**Authors:** Daniela Galicia-Diez Barroso, Luis David Abeijón-Malvaez, Gloria Alejandra Moreno Altamirano, María Esther Josefina Irigoyen-Camacho, Tracy L. Finlayson, Socorro Aída Borges-Yáñez

**Affiliations:** 1 Master’s and Doctorate Program in Medical, Dental, and Health Sciences (PMDCMOS), National Autonomous University of México (UNAM), Mexico City 04510, Mexico; dgalicia@fo.odonto.unam.mx; 2 School of Dentistry, Technological University of México (UNITEC), Mexico City 11320, Mexico; luis_abeijon@my.unitec.edu.mx; 3 Department of Public Health, School of Medicine, National Autonomous University of México (UNAM), Mexico City 04510, Mexico; gamoreno@unam.mx; 4 Health Care Department, Metropolitan Autonomous University (UAM-Xochimilco), Mexico City 14387, Mexico; meirigo@correo.xoc.uam.mx; 5 Division of Health Management and Policy, School of Public Health, San Diego State University, San Diego, CA 92182, USA; tfinlays@sdsu.edu; 6 Department of Dental Public Health, Graduate Studies and Research Division, School of Dentistry, National Autonomous University of México (UNAM), Mexico City 04510, Mexico

**Keywords:** adolescent, oral health, dental health services, psychosocial factors, access, Andersen model

## Abstract

Many factors contribute as facilitators of or barriers to adolescents’ use of dental services. Guided by the expanded Andersen model for dental service utilization, the aim of this study was to identify factors associated with the use of dental services among adolescents ages 12–16 in south Mexico City (*n* = 247). Adolescents answered a questionnaire on predisposing factors (age and gender), enabling factors (socioeconomic status, oral health support, parental years of education, and previous dental treatments), and psychosocial and behavioral factors (attitudes towards oral health; knowledge of gingivitis; alcohol, drug, and tobacco use; and depressive symptoms), and they underwent a visual clinical exam to determine their need factors (caries and gingivitis). The adolescents reported whether or not they had attended a dental visit in the last year for any reason. Multiple logistic regression was used to evaluate these factors. Having oral health support increased the odds of a dental visit by 2.69 (95% CI = 1.24–5.84). Previous dental treatment increased the odds of a dental visit by 2.25 (95% CI = 1.12–4.52). The presence of depressive symptoms reduced the odds of a dental visit by 4% (OR = 0.96, 95% CI = 0.94–0.99). Enabling and psychosocial factors of oral health support and previous dental treatment were positively associated with the utilization of dental services, while depressive symptoms were negatively associated.

## 1. Introduction

In Mexico, 84% of dental care is private [1], including dental offices, university teaching clinics, and pharmacies, where each individual must pay for their care. The remainder comprises public services, whose beneficiaries are mostly government employees and their families. Primary preventive care and restorative treatments are provided, including dental prophylaxis, scaling, resin and amalgam restorations, pit and fissure sealants, and tooth extractions [2]. In Mexico, there are scant data on the utilization of dental services by adolescents. The most recent data from a national survey in 2008 reported that 65.20% of adolescents in junior high school had never used dental services, the main reason being that they did not need them. However, a large portion who used dental services (28.20%) did so because they were in pain [3]. A study conducted in Mexico City among high school adolescents in 2014 reported that 46.90% had visited the dentist in the previous 12 months. Regarding the main reasons for attending, it was found that 55.80% went for preventive care and 32.70% went for restorative care. Regarding the barriers preventing attendance, 58.10% reported that it was expensive and 29.10% reported that they did not attend because they did not need it [4].

The main oral diseases in adolescents are caries and gingivitis [5,6]. These diseases do not cause pain in the early stages, which means that adolescents are generally not very aware of these diseases and have little interest in preventing them. Even though dental eruption is completed in adolescence and these diseases may be present, the adolescents prioritize improving their appearance and dental aesthetics due to peer interaction and social acceptance [7]. Although parents are responsible for their children’s oral health, it is at this stage that adolescents begin to develop their autonomy, self-image, perception of disease, and responsibility for their own health [8,9]. Visits to the dentist depend not only on the decision of the parents, but also on the perceived needs of the adolescents.

Different factors have been associated with the use of dental services, and several models have been developed to explain this phenomenon. One of the most widely used models is the behavioral model of healthcare utilization proposed by Andersen and Newman [10], which proposes that the utilization of services is determined by social, individual, and healthcare system factors. At the individual level, traditional predisposing factors include sociodemographic characteristics, enabling factors include financial aspects, and need factors include perceptions about health status [10].

Years later, Bradley et al. [11] proposed the inclusion of psychosocial factors in the model, since they consider that the beliefs of individuals are not sufficient—as mentioned in the Andersen Model, as they may not adequately capture the breadth of psychosocial factors and thus explain the factors associated with long-term service utilization. Psychological factors such as depression, stress, anxiety, self-esteem, and self-perception of general and oral health may help further explain people’s decisions to utilize different health services during adolescence.

Most service utilization models have been applied to adult populations; there are few studies that identify factors associated with adolescent’s use of health services. The aim of this study was to use the expanded Andersen model to identify factors associated with the utilization of dental services by junior high-school adolescents from the south of Mexico City. Identifying factors associated with the utilization of dental services will allow us to recognize the areas of opportunity to promote regular dental-care use, and to promote better long-term oral health. Understanding and influencing adolescent’s dental care behavior can set the foundation for a lifetime of good oral health practices [12]. By promoting good dental care behavior in adolescents, public health initiatives can potentially reduce the economic burden associated with treating extensive dental issues later in life [13].

## 2. Materials and Methods

**Figure 1:**
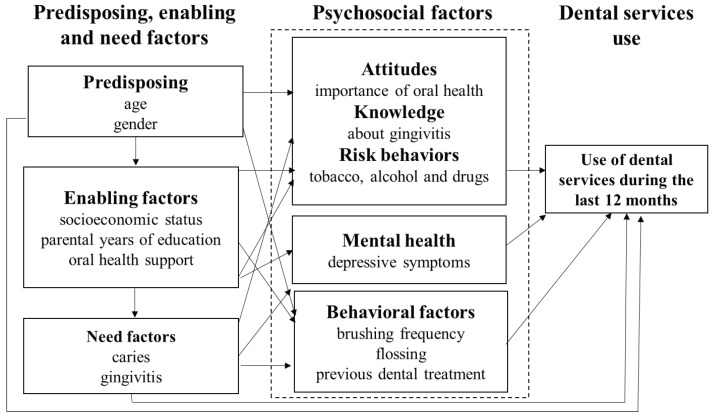
Variables considered for the expanded Andersen model for health services utilization.

The design of this study was cross-sectional, with a study population of 430 students enrolled in a secondary school located in the south of Mexico City during the year 2019. According to statistical data from the Federal Education Authority in Mexico City [14], for the 2018–2019 school year in Tlalpan, a county where Secondary School Num. 281 is located, 30,790 students were enrolled in public high schools. The sample was non-probabilistic and consisted of the total number of students who agreed to participate in the study (*n* = 247). However, the sample size was estimated to identify differences in the social support of students with and without caries. This resulted in 228 adolescents, to achieve a power of 85.20%. The statistical program PASS 15 Power Analysis and Sample Size Software (2017) was used (NCSS, LLC., Kaysville, UT, USA). Students between 12 and 16 years old that wanted to participate and had an informed consent form signed by their parents were included. This study was approved by the Research and Ethics Committee of the Faculty of Dentistry of the National Autonomous University of Mexico (UNAM), approval number CIE/0910/11/2018. The confidentiality and security of the data provided were guaranteed to the participants. A self-administered questionnaire was designed to collect information on the variables of interest according to Andersen’s expanded model (Figure 1) (Supplementary Materials) [11].

The predisposing factors were age (years) and gender (male/female). The enabling factors were socioeconomic status (0–283 points) [15], parental years of education (0–18 years), and oral health support, meaning the adolescent has a person to take them to the dentist (with/without support). The need factors were the presence of caries (yes/no), the presence of gingivitis, and the clinical examination showing bleeding upon probing (yes/no). The psychosocial and behavioral factors were depressive symptoms (0–60 points); tobacco, alcohol, or drug use at least once in their life (yes/no); the frequency of brushing (number of times per day); the use of dental floss (yes/no); knowledge related to gingivitis (adequate/not adequate); attitude towards the importance of oral health (adequate/inadequate); and the presence of previous dental treatment (yes/no). The main variable asked about was the use of dental services during the last 12 months (yes/no).

Additional questions asked about were the type of service used (public/private, teaching clinics/pharmacies), the frequency of their use of services (number of visits during the last 12 months), the person who paid for the services (adolescent/family members), and the reason for using dental services (dental check-up/tooth cleaning/pain/restorative treatment/orthodontic treatment).

Socioeconomic status (SES) was measured using the Mexican Association of Market Intelligence and Opinion Agencies AMAI index (Asociación Mexicana de Agencias de Inteligencia de Mercado y Opinión in Mexico) [16]—a scale validated in Mexican population. The scale ranges from 0 to 283 points, and the higher the score obtained, the higher the socioeconomic level is considered to be. The oral health support variable was assessed using the question “there is a person who takes me to the dentist when I need it”. The response scale was a Likert-type scale rated from 1 to 5, with answers ranging from never to always. The students who obtained a value of 3 or more were classified as having oral health support and those with 1 to 2 points were classified as having no support. To determine the presence of depressive symptoms, the version of the Scale of the Center for Epidemiological Studies aimed towards adolescents (CES-D) was used, which consists of twenty items [17]. This is a validated scale in Mexican adolescents, where the higher the score, the greater the presence of depressive symptoms; the scale ranges from 0 to 60 points. Knowledge of gingivitis was measured using the statement “bleeding when brushing is a sign of gum disease”; students who answered that the statement was true were considered to have adequate knowledge of gingivitis, and those who answered that the statement was false or did not know were considered to have inadequate knowledge. For the variable of attitude toward the importance of oral health, participants were asked to list the following activities in order of importance: 1 = taking care of my personal appearance, 2 = taking care of my general health, 3 = taking care of my oral health, 4 = taking care of my interpersonal relationships, and 5 = taking care of my grades. The adolescents who ranked taking care of their oral health in first place were considered to have an adequate attitude.

Regarding clinical variables, the International Caries Detection and Assessment System (ICDAS) was used to classify the presence of dental caries, with code 3 indicating the presence of caries [18]. The restorations component of the ICDAS was used to classify the variable of previous dental treatment through the presence of at least one tooth with sealants, amalgam, temporary fillings, or resin restorations. Variable gingivitis was evaluated using the gingival margin bleeding index [19], where the categories of blood spot and excessive bleeding were considered to indicate the presence of bleeding on probing. It is important to mention that a previous standardization of the examination by an expert in the ICDAS index was performed in ten adolescent volunteer patients, obtaining a kappa value of 0.81. A kappa value of 1.0 was obtained for the gingival margin bleeding index. Standardization of the recorders was also performed to ensure correct recording of the data obtained.

The clinical examination was performed on 204 students who agreed to the oral examination. Some explanations regarding the 43 students who did not want to participate included they were afraid that the oral exam would hurt and some students stopped attending school. The oral exam was performed in a classroom provided by the school, with prior disinfection of the furniture used, asking each of the students to lie down on a table, according to the recommendations of the World Health Organization (WHO) [20]. One standardized examiner with one recorder participated in the oral exam. To determine the students’ ICDAS index, each participant was provided with a toothbrush and toothpaste and asked to brush their teeth, then the surfaces were dried using a triple syringe in order to evaluate the dental surface and determine the codes. Next, the index of bleeding at the gingival margin was recorded using a PCP.11 probe, which was inserted 2 mm into the gingival margin of each tooth, moving from distal to mesial, waiting 30 s to check for the presence of bleeding.

Data were analyzed using STATA 14.2 software. For statistical analysis, measures of central tendency and dispersion were obtained for all variables (age, SES, years of parents’ education, depressive symptoms, brushing frequency, and frequency of utilization of dental services during the last 12 months), and proportions were obtained for the qualitative variables (gender; oral health support; presence of caries; presence of bleeding on probing; use of tobacco, alcohol, or drugs once in life; use of dental floss; knowledge related to periodontal disease; attitude toward the importance of oral health; presence of previous restorative treatment; use of dental services during the last 12 months; type of service used; frequency of use of the services; person who paid for the services; and reason for using dental services). The dependent variable was the use of dental services during the last 12 months, and the dependent variables were the predisposing, enabling, need, psychosocial, and behavioral factors. Subsequently, a bivariate analysis was performed to determine the association between the use of dental services during the last 12 months and the variables identified in the expanded Andersen model. For the multiple-variable analysis, logistic regression was performed considering the variables for which *p* < 0.25 in the bivariate analysis and variables with theoretical significance. The variable of years of parental education was eliminated because it caused 43 missing values. The model included the variables that best fit the Hosmer–Lemeshow goodness-of-fit test.

## 3. Results

### 3.1. Use of Dental Services

The study group consisted of 247 students. It was found that 125 (50.61%) of the adolescents had used dental services in the last 12 months; of these, 36 (28.80%) reported having used public services, while 70 (56%) reported having used private services, and 19 (15.20%) reported having used services of teaching clinics and pharmacies’ offices. Regarding the main reasons for using dental services, 70 (56%) reported having used them for dental check-ups and cleaning, 29 (23.20%) used them due to pain, 19 (15.20%) reported having used them for restorative treatment, and seven (5.60%) reported having used them for orthodontic treatment. Adolescents reported that all the treatments performed through private services were paid for by their family members.

### 3.2. Predisposing, Enabling, and Need Factors

The mean age of this population was 13.51 ± 1.09 years, and 51.82% were female. In terms of SES, a mean of 164.76 ± 53.96 points was observed using the AMAI scale, which has a maximum score of 283 points, so it could be considered the middle of the scale. The mean years of education of the fathers was 9.44 ± 3.59 years, and for the mothers, it was 9.50 ± 3.69 years, which in Mexico is equivalent to a high-school education. The majority 182 (73.68%) of the adolescents reported having oral health support, meaning they had someone to take them to the dentist when they needed it. Related to need factors, it was observed that more than half (59.31%) of the adolescents had at least one tooth with caries and nearly all (96.57%) had at least one tooth with bleeding on probing.

### 3.3. Psychosocial Factors and Behavioral Factors

The mean depressive symptoms score was 20.14 ± 11.06 points, with 60.32% having depressive symptoms. Regarding the use of tobacco, drugs, and alcohol, respectively, 27.13%, 13.36%, and 48.18% of the adolescents reported having used these substances at some point in their lives. The median frequency of brushing was two [IQR = 2–3] times per day. Only 86 (34.82%) of the adolescents reported flossing. Few adolescents (23.89%) were knowledgeable about gum bleeding as a sign of gum disease (gingivitis), and only 17% had a good attitude towards the importance of taking care of their oral health. It was observed that about one third (32.35%) of the adolescents had previously received dental treatment.

### 3.4. Bivariate Associations

A higher percentage of females reported the use of dental services during the last 12 months, compared to males (58.40% vs. 41.60%, *p* = 0.04). The mean SES was higher in those adolescents who attended the dentist compared to those who did not attend (171.56 ± 52.13 vs. 157.78 ± 55.11, *p* = 0.04). Adolescents with oral health support attended in greater proportion than those without support (85.60% vs. 14.40%, *p* = 0.01). Adolescents who had used dental services during the last 12 months (18.38 ± 10.38) had fewer depressive symptoms that those who had not used dental services (21.95 ± 11.49, *p* = 0.01). The proportion of adolescents who had used dental services during the last twelve months was higher among adolescents who had never used tobacco in their lives (80% vs. 20%, *p* = 0.01). Those adolescents who flossed attended the dentist less in the last twelve months than those who did not floss (47.20% vs. 52.80%, *p* = 0.01). A higher percentage of adolescents with presence of previous dental treatment reported a dental visit during the last 12 months, compared with those adolescents without previous dental treatment (42.45% vs. 57.55%, *p* = 0.01) (Table 1).

The variables included in the logistic regression model to predict the use of dental services in the last 12 months were age, gender, SES, oral health support, presence of at least one tooth with caries, depressive symptoms, tobacco use once in life, brushing frequency, flossing, attitude towards the importance of oral health, and presence of previous dental treatment. A total of 204 adolescents were included in the model; it was adjusted appropriately (Hosmer–Lemeshow = 197.79, *p* = 0.37) and explained 17.46% of the variability. In this model, having oral health support increased the odds of using dental services during the last 12 months by 2.69 (95% CI = 1.24–5.84) compared to not having oral health support. Previous dental treatment increased the odds of having used dental services during the last 12 months by 2.25 (95% CI = 1.1–4.52) compared to not having dental treatment. Similarly, each unit increase in the CES-D scale is associated with a 4% decrease in the odds of having used dental services during the last 12 months (OR = 0.96, 95% CI = 0.94–0.99) (Table 2).

## 4. Discussion

The aim of this study was to use the expanded Andersen model to identify the factors associated with the use of dental services. Enabling and psychosocial factors including oral health support and the presence of previous dental treatment were also associated with adolescents’ use of dental services during the last 12 months. Depressive symptoms were negatively associated with dental visits.

The predisposing factors, age and gender, did not show an association in this study, in contrast to studies conducted in the United States [21], Chile [22], and Brazil [23], male adolescents were observed to be less likely to use dental services, and late adolescents (15–17 years) were less likely to use services compared to early adolescents (11–14 years). These differences may be due to the fact that the population of their study included adolescents from all stages of adolescence, while this study only included early adolescents.

Similarly, no association between socioeconomic status and parental years of education was observed in this population, in contrast to studies from the United States [21], Chile [22], and Brazil [23], where parental education and socioeconomic status were identified to be enabling factors associated with the use of dental services. This difference could be due to the way in which the other studies classified parental education and socioeconomic status; the educational systems of other countries may also be of better or worse quality in terms of health promotion. It may also be due to the fact that the systems in other countries include greater coverage for adolescents, which is not the case in Mexico, where public services have a limited range of services, including only fillings, extractions, and preventive care, limiting the use of specialized treatments, which can generate a barrier to the use of services. Additionally, the culture of the different countries may affect these results, as more or less importance may be assigned to the use of services in different countries. The study from Chile evaluated dental attendance over 12 months and its population included adolescents and young adults.

The enabling factor of “social support in oral health” more than doubled the odds of adolescents using dental services compared to those who did not have oral health support. This association was very similar to what was reported in studies from Brazil [24] and the United States, conducted among Latino children, adolescents, and migrants [25,26]. Emotional support has been shown to help overcome fears and reduce stress in adult patients during dental care [27]. Social support in oral health is an important factor in dental visits and oral health. Adolescents depend on this support to a great extent, since it may be associated with their families attending these visits regularly [28].

In this study, no association was observed between the need factors (dental caries and gingivitis) and the use of services during the last 12 months, similar to the results of the Brazilian study [23] where no association was observed between the presence of gingivitis and periodontal pockets. However, the study from China [29] found that the severity of caries was associated with the use of dental services; this association could be due to the way the severity of caries was classified and the fact that the adolescents were in late adolescence. Early adolescence is a period when individuals, who have made the transition from primary to permanent dentition, have teeth with earlier stages of dental caries, so most of them may have asymptomatic disease and therefore may perceive that it is not necessary to go to the dentist because they do not have any pain. Similarly, early adolescents may perceive gingival bleeding as the result of “too hard” or traumatic tooth brushing and may not associate it with gingival inflammation. In this study, 97% of participants had gingival bleeding, which may explain why no association was found between gingivitis and dental visits.

With respect to the psychosocial and behavioral factors, this study did not find an association between the use of dental services and the frequency of brushing, contrary to what was observed in a study conducted among adolescents in China, which identified that not brushing or doing so occasionally increased the probability of not using dental services [29]. This is similar to a study in an Iranian population of patients aged 15 to 64 years, where it was observed that there was an association between brushing frequency and dental visits. Those who did not brush their teeth were less likely to visit the dentist than to those who brushed once, and those who brushed twice or more [30]. This association may be due to the way in which they categorized the frequency of brushing, in addition to the fact that these studies mention that brushing teeth less frequently leads to worse oral health conditions, which causes adolescents to seek care because they have medical needs. Similarly, the observed differences in association may be due to the existence of a social desirability bias, where adolescents may report a higher frequency of brushing. This is because high levels of dental biofilm were observed during the oral examination, which may be related to poor brushing technique and eating between meals.

Regarding the psychosocial factor depressive symptoms, it was observed in this study that as depressive symptoms increase, the probability of using dental services decreases, similar to what was reported in a study from Finland [31] on an older (adult) Finnish population where women with depressive symptoms took longer to visit the dentist than men. Similarly, in a population of older German adults, the presence of depressive symptoms was reported to be an unfavorable factor for the use of dental services [32]. It is worth noting that the studies that found this association were conducted in populations of older adults; there is no evidence of this association in populations of adolescents. Depressive symptoms may affect the oral health of individuals by influencing their self-perception and can negatively modifying their behaviors and habits, leading to the development of diseases such as caries and periodontal disease [33].

Regarding the psychosocial factor tobacco use once in life, a survey conducted in the United States [34] found that adult smokers reported fewer dental visits than nonsmokers. On the other hand, among persons aged 18 years or older, a higher frequency of dental visits in the past year was found among people who never smoke than in current smokers. The results of this study report that smokers visit the dentist less frequently than adolescents who have never smoked, which is consistent with what has been reported in studies from the United States [34] and Canada [35]. Although tobacco use is a risk factor for oral diseases such as dental caries, periodontal disease, and cancer, dental visits by smokers are limited to special situations and are not a regular practice for prevention, diagnosis, or oral treatment, so smokers use dental services less [34].

One of the limitations of the study is that, as a cross-sectional study, a temporal bias must be taken into account, since it is difficult to distinguish the temporal order of the factors in the Andersen model in the use of dental services. Another limitation is the selection of the population, which makes it difficult to extrapolate to other groups of adolescents or to identify causal relationships; however, there are large sectors in Mexico that share similar socio-economic conditions and availability of services as the adolescents in the sample. Among the recommendations for future studies, it would be important to perform a study that includes adolescents from all Mexican regions and all socioeconomic levels, for example adolescents who attend school and adolescents who cannot attend school, or live on the streets. Future studies would also benefit from including variables such as quality of life and self-perception of oral health. The way adolescents perceive themselves may have an impact on their use of dental services. It is also of interest to look more closely at the habits of parents, as they have a major impact on adolescents’ attitudes and practices regarding the use of dental services and the perception of oral disease during adolescence. Investing in the oral health of adolescents and promoting healthy habits will contribute to better oral health throughout life, resulting in less tooth loss and therefore better overall health and quality of life.

The information collected in this study contributes to explaining which factors of the Andersen model contribute to the utilization of dental services in the Mexican adolescent population at an early stage, since there are few studies that examine this association.

Because most oral health studies and public policies focus on children, the adolescent population does not have specific oral health strategies. As a result, adolescents acquire incorrect knowledge and inappropriate behaviors that affect their oral health. With the information from this study, preventive programs can be designed to improve oral care habits and prevent chronic diseases such as periodontal disease and dental caries, focusing on psychosocial factors, which were observed to be very important in this population. Similarly, great attention should be paid to factors such as mental health, since this study observed that depressive symptoms have an important weight and can affect dental attendance factors, as can motivational factors. A comprehensive approach is required in adolescents, and variables such as self-esteem, self-efficacy, quality of life, and self-perception of oral health should be considered. For subsequent studies evaluating oral care behaviors, such as tooth brushing and dental visits, it is important to consider not only the frequency of these behaviors, but also the quality with which they are performed.

## 5. Conclusions

Enabling and psychosocial factors such as social support in oral health and the presence of previous dental treatment were positively associated with the utilization of dental services, while depressive symptoms were negatively associated with their utilization.

The use of the expanded Andersen model helps identify social factors influencing the utilization of dental services in order to create health strategies or policies to ensure access to services during this important stage of life, so that adolescents can acquire healthy habits at an early stage. This can serve as a basis for maintaining healthy practices that can be developed throughout all stages of life, resulting in a better oral health status and a better quality of life.

## Figures and Tables

**Table 1:** Bivariate analysis of the variable of using dental services and the predisposing, enabling, need, and psychosocial factors.

Variable	Utilization of Dental Services in the Last 12 Months			
	No (*n* = 122)	Yes (*n* = 125)	Total (*n* = 247)	*p*
**Predisposing factors**				
Age, mean (SD)	13.56 (1.15)	13.45 (1.02)	13.51 (1.09)	0.46 *
Gender	*n* (%)	*n* (%)	*n* (%)	
Male	67 (54.92)	52 (41.60)	119 (48.18)	0.036 **
Female	55 (45.08)	73 (58.40)	128 (51.82%)	
Total	122 (100)	125 (100)	247 (100)	
**Enabling factors**				
Socioeconomic status				
Mean (SD)	157.78 (55.11)	171.56 (52.13)	164.76 (53.96)	0.045 *
Mothers’ years of education	No (*n* = 113)	Yes (*n* = 115)	Total (*n* = 228)	
Mean (SD)	9.06 (3.81)	9.93 (3.53)	9.50 (3.69)	0.07 *
Fathers’ years of education	No (*n* = 103)	Yes (*n* = 103)	Total (*n* = 206)	
Mean (SD)	8.96 (3.29)	9.92 (3.83)	9.44 (3.59)	0.055 *
Oral health support				
With support	75 (61.48)	107 (85.60)	182 (73.68)	0.001 **
Without support	47 (38.52)	18 (14.40)	65 (26.32)	
Total	122 (100)	125 (100)	247 (100)	
**Need factors**				
Caries	Yes (*n* = 98)	No (*n* = 106)	Total (*n* = 204)	
Yes	54 (55.10)	67 (63.21)	121 (59.31)	0.24 **
No	44 (44.90)	39 (36.79)	83 (40.69)	
Total	98 (100)	106 (100)	204 (100)	
Gingivitis	Yes (*n* = 98)	No (*n* = 106)	Total (*n* = 204)	
Yes	95 (96.94)	102 (96.23)	197 (96.57)	0.78 **
No	3 (3.06)	4 (3.77)	7 (3.43)	
Total	98 (100)	106 (100)	208 (100)	
**Psychosocial and behavioral factors**				
Depressive symptoms				
Mean (SD)	21.95 (11.49)	18.38 (10.38)	20.14 (11.06)	0.012 ***
Median (IQR)	20.50 (19)	16 (15)	17 (28)	
Alcohol				
Yes	65 (53.28)	54 (43.20)	119 (48.20)	0.11 **
No	57 (46.72)	71 (56.80)	128 (51.82)	
Total	122 (100)	125 (100)	247 (100)	
Tobacco				
Yes	42 (34.43)	25 (20.00)	67 (27.13)	0.01 **
No	80 (65.57)	100 (80.00)	180 (72.87)	
Total	122 (100)	125 (100)	247 (100)	
Drugs				
Yes	20 (16.39)	13 (10.40)	33 (13.36)	0.17 **
No	102 (83.61)	112 (89.60)	214 (86.64)	
Total	122 (100)	125 (100)	247 (100)	
**Psychosocial and behavioral factors**				
Brushing frequency				
Mean (SD)	2.36 (0.99)	2.49 (0.78)	2.42 (0.89)	0.10 ***
Median (IQR)	2 (1)	2 (1)	2 (1)	
Flossing				
Yes	27 (22.13)	59 (47.20)	86 (34.82)	0.001 **
No	95 (77.87)	66 (52.80)	161 (65.18)	
Total	122 (100)	125 (100)	247 (100)	
Knowledge of gingivitis				
Not suitable	92 (75.41)	96 (76.80)	188 (76.11)	0.79 **
Suitable	30 (24.59)	29 (23.20)	59 (23.89)	
Total	122 (100)	125 (100)	247 (100)	
Attitude towards the importance of oral health				
Adequate	16 (13.11)	26 (20.80)	42 (17.00)	0.10 **
Inadequate	106 (86.89)	99 (79.20)	205 (83.00)	
Total	122 (100)	125 (100)	247 (100)	
Presence of previous dental treatment				
Yes	21 (21.43)	45 (42.45)	66 (32.35)	0.001 **
No	77 (78.57)	61 (57.55)	138 (67.65)	
Total	98 (100)	106 (100)	204 (100)	

* *t*-Test, ** Chi2 *** Mann–Whitney U Test. Depressive symptoms, CSD-20 Scale; SES, AMAI index.

**Table 2:** Logistic regression model results.

Variables	Crude OR (95% CI)	*p*	Adjusted OR (95% CI)	*p*
**Predisposing factors**				
Age	0.91 (0.72–1.14)	0.43	1.03 (0.75–1.41)	0.86
Gender				
Male	1			
Female	1.71 (1.03–2.83)	0.03	1.39 (0.73–2.66)	0.31
**Enabling factors**				
SES	1.004 (1.01–1.01)	0.04	1.01 (0.99–1.01)	0.09
Oral health support				
No	1			
Yes	3.72 (2.01–6.91)	0.001	2.69 (1.24–5.84)	0.012
Mothers’ years of education	1.07 (0.99–1.15)	0.07	-	-
Fathers’ years of education	1.07 (0.99–1.17)	0.05	-	-
**Need factors**				
Caries				
No	1			
Yes	1.39 (0.79–2.45)	0.24	1.78 (0.91–3.46)	0.08
**Psychosocial factors**				
Depressive symptoms	0.97 (0.95–0.99)	0.012	0.96 (0.94–0.99)	0.03
Tobacco use once in life				
Yes	1			
No	2.1 (1.18–3.73)	0.012	2.19 (0.99–4.86)	0.05
Alcohol use once in life				
Yes	1			
No	1.49 (0.91–2.48)	0.11	-	-
Drug use once in life				
Yes	1			
No	1.69 (0.79–3.57)	0.17	-	-
Drug use once in life				
Yes	1			
No	1.69 (0.79–3.57)	0.17	-	-
Brushing frequency	1.18 (0.89–1.57)	0.23	1.24 (0.86–1.80)	0.24
Flossing				
No	1			
Yes	3.14 (1.81–5.47)	0.001	1.87 (0.94–3.71)	0.07
Attitude towards the importance of oral health				
Inadequate	1			
Adequate	1.74 (0.88–3.43)	0.11	1.69 (0.69–4.17)	0.24
Previous dental treatment				
No	1			
Yes	2.7 (1.46–5.02)	0.002	2.25 (1.12–4.52)	0.02

## Data Availability

The datasets analyzed during the current study are available from the corresponding author on reasonable request.

## Supplementary Materials

The following supporting information can be downloaded at: https://www.mdpi.com/article/10.3390/healthcare11243159/s1, File S1. Questionnaire.
