# Supplementary material for: Using the Expanded Andersen Model to Determine Factors Associated with Mexican Adolescents’ Utilization of Dental Services
Source: Healthcare (Basel). 2023 Dec 13;11(24):3159. doi: 10.3390/healthcare11243159 (PMC10743091; doi:10.3390/healthcare11243159)
Supplement: Supplementary file 1 [file healthcare-11-03159-s001.zip › healthcare-2708157-supplementary.pdf]

MAESTRÍA Y DOCTORADO en Ciencias Médicas Odontológicas y de la Salud

UNIVERSIDAD NACIONAL AUTÓNOMA DE MÉXICO

FACULTAD DE ODONTOLOGÍA UNAM 1904

Instructions: Read the question carefully and place in the box the number that corresponds to your answer. The information you provide will be kept confidential and will be used only for statistical purposes. Thank you very much for your participation!

Date: Year ☐☐ Month ☐☐ Day ☐☐ Identification Number ☐☐☐☐

General information

Name: ____

Date of birth Year/Month/Day ☐☐☐☐☐☐

Grade: 1= 1st year of junior high school ☐

2= 2nd year of junior high school

3= 3rd year of junior high school

Age ☐☐ years

Sex 1= Male, 2= Female ☐

Occupation 1= Student, 2= Employee, 3= Student and employee ☐

Socioeconomic information

1. Thinking about the person who supports your household (the head of household), what was the last year of school he/she passed? Who is this person? ____________________ ☐

1= Did not study, 2= Pre-school, 3= Incomplete primary, 4= Complete primary, 5= Incomplete secondary, 6= Incomplete secondary, 7= Incomplete secondary, 8= Incomplete secondary, 5= Incomplete junior high school, 6= Complete high school, 7= Incomplete high school, 8= High school complete, 9= Bachelor's degree incomplete, 10= Bachelor's degree complete, 11= Graduate degree, 99= Don't know/no answer.

2. How many bathrooms with shower and toilet are there in your home? ☐

3. How many cars or trucks do you have in your household, including enclosed trucks, or trucks with cabs or boxes? ☐

4. Without taking into account the mobile connection that you may have from a cellular phone ☐

Does your household have internet? 0= No, 1= Yes

5. Of all the persons with 14 years old, or older living in your household, how many worked during the last month? ☐

6. In your household, how many rooms are used for sleeping, not counting hallways and bathrooms? ☐

Information about your parents

1. What was the last year of schooling your father completed?
1= Did not study, 2= Incomplete elementary school, 3= Completed elementary school, 4= Incomplete junior high school, 5= Junior high school complete, 6= Commercial career, 7= Technical career, 8= High school incomplete, 9= High school complete, 10= Bachelor's degree incomplete, 11= Bachelor's degree complete, 12= Graduate degree, 99= Don't know / didn't answer.

2. What was the last year of school your mother completed?
1= Did not study, 2= Incomplete elementary school, 3= Completed elementary school, 4= Incomplete junior high school, 5= Junior high school complete, 6= Commercial career, 7= Technical career, 8= High school incomplete, 9= High school complete, 10= Bachelor's degree incomplete, 11= Bachelor's degree complete, 12= Graduate degree, 99= Don't know / didn't answer.

3. What is your father's occupation?
1= Unemployed, 2= Employed, 3= Retired, 4= Trader, 5= Tradesman, 6= Other:_____________, 9= Don't know

4. What is your mother's occupation?
1= Unemployed, 2= Employee, 3= Retired, 4= Trader, 5= Trades, 6= Homemaker, 7= Other:________, 9= Don't know.

Oral health information

1. Have you ever been to the dentist in your life? 0= No, 1= Yes, 9= Don't know

If you have not been to the dentist, please skip to question 7 and put the number 9 in all the boxes.

2. In the last twelve months, have you been to the dentist? 1= Yes, 0= No, 9= Not Applicable

3. If you went to the dentist in the last twelve months, what was the reason?

1= Revision, 2= Cleaning, 3= Pain, 4= Restorative treatment (resins, crown), 5= Trauma, 6=Wisdom teeth, 7= Brackets, 9= Not applicable, 10= Other______________

4. How many times did you visit the dentist in the last twelve months?

5. What type of dental service did you go to? 1= IMSS, ISSSTE, Seguro popular, 2= Private clinic 3= Teaching clinic (e.g. UNAM, UAM, ULA, other), 4= Pharmacies, 9= Not applicable

6. Who paid for your treatment? 1= Myself, 2= My parents, 3= A family member, 4= Someone else, 5= It was free, 9= Not applicable

7. If you have not visited a dentist in the last twelve months, what was the reason?

1= I don't need it, 2= - I do not have anyone to take me, 3= It's expensive, 4= It scares me, 5= Other: ___________, 9= Not Applicable

8. Do you have to visit the dentist at least once a year, even if you have no problems or pain in your teeth?
0= False, 1= True, 2= Don't know

9. Do you brush your teeth?
0= No, 1= Yes, 2= Sometimes

10. In case you do not brush your teeth, what is the main reason?
1= I don't have a toothbrush, 2=I don't know how to brush my teeth, 3=I feel lazy, 4=I forget, 5=I don't have time, 6=I don't consider it important, 9=Not applicable

11. If you brush your teeth, how many times a day do you brush your teeth?

12. Which brushing do you consider to be the most important?
1= In the morning, 2= After lunch, 3= Before bedtime, 4= Don't know

13. Which brushing do you do most of the time?
1= In the morning, 2= In the afternoon, 3= In the evening, 4= After meals, 5= Don't know

14. What items do you use to brush your teeth?
1= Water, 2= Toothpaste, 3= Toothpaste and water, 4= None

15. How long do you think the brushing should take?
1= One minute, 2= Two minutes, 3= Three minutes, 4= Don't know

16. How often should you change your toothbrush?
1= One to three months, 2= Four to six months, 3= Seven to twelve months, 4= More than one year, 5= Don't know.

17. What movements do you make when brushing your teeth with a toothbrush?
1= Horizontal, side to side movements, 2= Up and down movements,
3= Circular movements, 4= Don't know

18. Brushing your teeth can prevent cavities.
1= False, 2= True, 3= Don't know

19. Do you use any hygiene aids in addition to your toothbrush?
0= No, 1= Yes

20. In case you use any complement or auxiliary, which one do you use? 1= Dental floss, 2= Mouthwash, 3= Interdental brush, 4= Brush for braces, 5= Other: ______________
9= Not applicable

21. Do you floss your teeth?
0= No, 1= Yes, 2= Sometimes

If you do not use dental floss, please skip to question number 23 and place the number 9 in all boxes.

22. If you use it, making an estimate, how often do you floss your teeth?
1= once a day, 2= twice a day, 3= three times a day, 4= once a week, 5= once a month,
9= not applicable

→**23. If you do not floss, what is the reason?**
1= I don't have dental floss, 2= I don't know how to use it, 3= I don't think I would be able to use it, 4= I am too lazy, 5= I forget, 6= I don't have time, 9= Not applicable.

24. Flossing does not prevent tooth decay.
0= False, 1= True, 2= Don't know

25. How important is it for you to clean your teeth?
1= Not important at all, 2= Not very important, 3= Important, 4= Very important

26. Bacteria (germs) on teeth cause cavities.
0= False, 1= True, 2= Don't know

27. Natural teeth are not better than false or false teeth.
0= False, 1= True, 2= Don't know

28. Some types of food can cause cavities
0= False, 1= True, 2= Don't know

29. The use of fluoride prevents tooth decay.
0= False, 1= True, 2= Don't know

30. Not brushing your teeth is the cause of bad breath.
0= False, 1= True, 2= Don't know

31. Bleeding on brushing is not a sign of gum disease.
0= False, 1= True, 2= Don't know

32. Lack of oral hygiene cannot cause any disease.
0= False, 1= True, 2= Don't know

33. What is most important to you? Put in the boxes, in order of importance, the number of the following activities.
The first box will be the most important activity for you.
1= Taking care of my personal appearance, 2= Taking care of my general health, 3= Taking care of my oral health, 4= Taking care of my interpersonal relationships, 5= Taking care of my grades, 5= Taking care of my personal health, 5= Taking care of my personal relationships, 6= Taking care of my grades.

Next, you will be asked questions about smoking and drug use practices, please place the number that corresponds to your answer in the box.

1. Have you ever used any type of **drug in your life**? 0= No, 1= Yes

2. Do you **currently** use any type of drug? 0= No, 1= Yes

3. Have you **ever smoked in your life**? 0= No, 1= Yes

4. Do you currently **smoke**? 0= No, 1= Yes

If you do not currently smoke, please skip to the next section, please place the number 9 in all boxes.

3. **How many cigarettes do you smoke per day**?
Number of cigarettes

How long after waking up do you smoke your first cigarette? 1= First 5 minutes,
2= Between 6 and 30 minutes, 3= Between 31 and 60 minutes, 4= More than one hour, 9= Not applicable

5. **When do you smoke the most**? 1= In the mornings, 2= In the afternoons, 3= Nowadays I don't smoke anymore.

6. **Do you find it difficult to refrain from smoking in places where it is prohibited**? (Libraries, movie theaters, no smoking sections in restaurants? 0= No, 1= Yes, 9= Not applicable.

7. **Do you smoke even when you have to stay in bed because of illness**?
0= No, 1= Yes, 9= Does not apply.

8. Of the cigarettes you smoke during the day, **which is the most difficult for you to quit**?
1= The first one in the morning, 2= The one after lunch, 3= When you are under stress, 4= The one in the bathroom, 5= The one in the bathroom, 5= The one after lunch,
4= The one in the bathroom, 5= The one before bedtime, 9= Does not apply

→9. Have you ever consumed alcoholic beverages in your life? 0= No, 1= Yes 1.

10. Do you currently **consume alcoholic beverages**?
0= No, 1= Yes

If you haven't consumed alcoholic beverages, please skip to the next section. And circle the answer that corresponds 9 does not apply Next, you will be asked questions about alcoholism practices, of the following questions, mark with an X the box that best describes your answer to each question	**0**	**1**	**2**	**3**	**4**	**9**
**1. How often do you drink alcoholic beverages?**	Never	Once a month or less	2 to 4 times a month	2 to 3 times a week	4 or more times a week	Not applica ble
**2. How many alcoholic beverages do you usually consume in a normal drinking day?**	From 1 to 2	3 o 4	5 o 6	From 7 to 9	10 or more	Not applica ble
**3. How often do you drink 6 or more alcoholic beverages in a single day?**	Never	Less than once a month	Monthly	Weekly	Daily	Not applica ble
**4. How often in the course of the last year have you been unable to stop drinking once you had started?**	Never	Less than once a month	Monthly	Weekly	Daily	Not applica ble
**5. How often in the past year were you unable to do what you were expected to do because you had been drinking?**	Never	Less than once a month	Monthly	Weekly	Daily	Not applica ble
**6. How often in the past year have you needed to drink on an empty stomach to recover from heavy drinking the day before?**	Never	Less than once a month	Monthly	Weekly	Daily	Not applica ble
**7. How often in the past year have you had remorse or feelings of guilt after drinking?**	Never	Less than once a month	Monthly	Weekly	Daily	Not applica ble
**8. How often in the past year have you been unable to remember what happened the night before because you had been drinking?**	Never	Less than once a month	Monthly	Weekly	Daily	Not applica ble
**Have you or anyone else been injured because you had been drinking during the course of the year?**	No		Yes, but not in the last year.		Yes, in the last year	Not applica ble
**10. Has any family member, friend, doctor or health professional shown concern about your drinking or suggested that you stop drinking?**	No		Yes, but not in the last year.		Yes, in the last year	Not applica ble

Read carefully and indicate how you feel about each of the following sentences, circle the answer you most identify with. Only one answer per question should be marked.	Never	Few times	Sometimes	Most of the time	Always
1. There is a person who takes me to the doctor when I need it.	1	2	3	4	5
2. There is a person who takes me to the dentist when I need it.	1	2	3	4	5
3. My parents support me to practice activities that interest me.	1	2	3	4	5
4. My parents help me make decisions	1	2	3	4	5
5. My parents give me good advice	1	2	3	4	5
6. My parents help me find solutions to my problems.	1	2	3	4	5
7. My teachers recognize me when I do things right.	1	2	3	4	5
8. My classmates include me in their activities	1	2	3	4	5
9. My best friend explains things to me that I don't understand.	1	2	3	4	5
10. My best friend helps me when I need it	1	2	3	4	5
11. My best friend asks me if I need help.	1	2	3	4	5
12. There is one person who encourages me when I am in trouble.	1	2	3	4	5
13. There is a person with whom I can share my sorrows and joys.	1	2	3	4	5
14. I receive useful advice about important things in life.	1	2	3	4	5
15. There is a person who advises me on how to take care of my health.	1	2	3	4	5
16. My family gives me the help and support I need.	1	2	3	4	5
17. There is one person who is a source of comfort to me.	1	2	3	4	5
18. I can talk about my problems with my family	1	2	3	4	5
19. My best friend listens to me when I have problems.	1	2	3	4	5
20. My parents show me that they are proud of me.	1	2	3	4	5
21. My parents understand me	1	2	3	4	5
22. My teachers care about me	1	2	3	4	5

The following statements describe ways people act or feel. Please read each one and circle the number of days you felt this way in **the last week**. (Check one answer per statement, if you are unsure make your best estimation).

**During the last week:**	**Number of days**			
1. I was bothered by many things that do not usually bother me.	0	1-2	3-4	5-7
2. I was not hungry, I had no appetite.	0	1-2	3-4	5-7
3. I felt that I could not get rid of the sadness even with the help of my family or friends.	0	1-2	3-4	5-7
4. Felt that he/she was as good as others	0	1-2	3-4	5-7
5. I had difficulty concentrating on what I was doing.	0	1-2	3-4	5-7
6. I felt depressed	0	1-2	3-4	5-7
7. I felt that everything I did cost me a lot of effort.	0	1-2	3-4	5-7
8. He saw the future with hope	0	1-2	3-4	5-7
9. I thought my life would be a failure	0	1-2	3-4	5-7
10. I was afraid	0	1-2	3-4	5-7
11. I slept without resting	0	1-2	3-4	5-7
12. I was happy	0	1-2	3-4	5-7
13. I talked less than usual	0	1-2	3-4	5-7
14. I felt lonely	0	1-2	3-4	5-7
15. I felt that people were unfriendly	0	1-2	3-4	5-7
16. I enjoyed life	0	1-2	3-4	5-7
17. He cried at times	0	1-2	3-4	5-7
18. I felt sad	0	1-2	3-4	5-7
19. I felt that others did not like me.	0	1-2	3-4	5-7
20. I could not move forward	0	1-2	3-4	5-7

Thank you very much for your participation
